# An innovative application of event structure analysis (ESA)

**DOI:** 10.1016/j.mex.2021.101256

**Published:** 2021-01-31

**Authors:** Jonathan Simões Freitas, Julio Cezar Fonseca de Melo, Mario Sergio Salerno, Raoni Barros Bagno, Vinicius Chagas Brasil

**Affiliations:** a Federal University of Minas Gerais (UFMG), Presidente Antônio Carlos Avenue, 6627 - Pampulha, Belo Horizonte, MG, Brazil; b Production Engineering Department Polytechnic School, University of São Paulo (USP), Av. Professor Luciano Gualberto, 1380 São Paulo, SP, Brazil

**Keywords:** Event structure analysis, ESA, Causal process tracing, Emergence, Critical events

## Abstract

This paper presents an innovative application of event structure analysis (ESA). The key improvements incorporated on the method are: (i) a robust system for coding events; (ii) the use of causal process tracing tests for inferring necessary connections; (iii) the combination of ESA with network analyses. Finally, we propose five types of analysis for event network models (*i.e.,* critical elements, critical associations, critical connections, critical specific happenings, and critical antecedents) and exemplify some of them in a causal case study about the process of capability construction for open innovation management in an Industrial Electronic Manufacturer.

• ESA can be combined with process-tracing tests to ground counterfactual causal inferences.
• ESA can be combined with network analysis to explore quantitative patterns in event structures.
• ESA is an outstanding method to conduct process research in management and engineering.

## Graphical abstract

**Figure:**
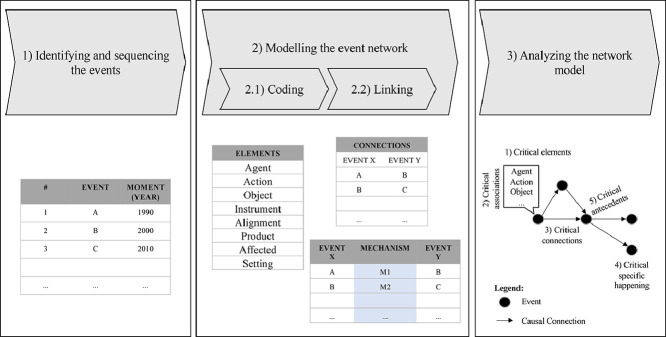

Specifications table

**Table:** 

Subject Area:	*Engineering*
More specific subject area:	*Portfolio and Project Management*
Method name:	*Event Structure Analysis (ESA)*
Name and reference of original method:	*Event Structure Analysis (ESA) was proposed at the end of the 1980s in Sociology*[23]*. Supplementing the seminal article, Corsaro, and Heise*[10]*, Griffin*[18]*and Griffin and Korstad*[19]*established the foundations of the method.*
Resource availability:	*ETHNO Software* (https://cs.uwaterloo.ca/~jhoey/research/ACTBackup/ESA/ESA.html) *VISONE Software (*www.visone.info*)*

## Method details

### Context

Event Structure Analysis (ESA) was proposed at the end of the 1980s [23]. Supplementing the seminal article, Corsaro, and Heise [10], Griffin [18] and Griffin and Korstad [19] established the foundations of the method. Given its originality and logical rigor, ESA contributed to establishing a new methodological category called ``formal qualitative analysis'' [20].

Several applications of the ESA procedures were made in the social sciences during the 1990s and 2000s. The release of the ETHNO software [22] in parallel with the first publications may have contributed to this diffusion (c.f. [1]). The fact is that, in fundamental methodological reviews on the analysis of processes and narratives, Mahoney [29] and Abell [2] were unanimous in recognizing ESA as the main analytical approach for intra-case study of events’ causal chains.

However, ESA was rarely applied in management-related fields [36], [37], [38]. Also, in general, these applications only replicated the basic procedures of the initial proposal of the method. None of them, for example, adopted the robust system for coding events later proposed by Heise and Durig [21]. Similarly, none of these papers explored the potential of the combination of ESA with network analyses, or with causal process tracing tests - which has been receiving a lot of attention in the field of comparative-historical methodologies in recent years (c.f. [5,^8^,^27^,30]).

## Explanation of the methodology

### Identifying and sequencing the events

Unlike variance research, which provides an explanation from the relationship between the dependent and independent variables, process theories are built from patterns extracted from a sequence of events [33]. Events can be defined as actions of a determined agent on a given object, at a specific moment of time [21] and may include decisions, meetings, conversations or even a simple, but explanatorily relevant, handshake (Langley, 1999). Thus, the methodological emphasis of process research lies on the historical explanation of an eventually remarkable macro-outcome that emerges over time.

Events can be identified based on semi-structured interviews with key participants of the studied phenomena in order to elicit the narrative of each interviewee. Researchers must interpret these narratives and come to a consensus regarding the set of events that summarizes the story. The discretization of the narrative - *i.e.,* of a “continuous discourse” - into distinct events is based on the attempt, by the researchers, to understand (*i.e.,* “verstehen”) the culture of the “natives” (c.f. [13]) - that is, of the selected interviewees. In fact, this understanding is fundamental, not only to distinguish the events adequately but also to describe them using a contextually meaningful language [10,23].

This abstraction from the original description (*i.e.,* the description by the interviewees themselves) consists, therefore, in a ``theoretical reading'' of the meaning of the event in the context of the structuring process under analysis. It involves the interpretation of the causal relevance of the elements of the ``concrete'' event in order to rephrase it as an ``abstract'' event [23,24].

Once the events have been properly identified and described, the researcher should sequence them in chronological order to be able to assess possible causal connections between them. After all, chronological antecedence is a necessary but insufficient condition for a historical explanation (c.f. [17,18], 1995; [19,31]). That is, although temporal precedence does not imply causation (i.e. some events may be entirely causally irrelevant to subsequent ones), it is obvious that an event cannot be caused by another event that succeeds it. Hence, sorting them chronologically reduces by half the upper limit of possible causal connections to be assessed [23]. Therefore, it is well advised that the events should be firstly sequenced in chronological order to - only then - be then analyzed in terms of causality. In conducting this analysis, researchers can search for accurate references to dates as a starting point for sequencing the events, using temporal conjunctions narrated by the interviewees or collecting support documents which corroborate the occurrence of a given event. Finally, it is also recommended to validate the final results with the interviewees.

## Modeling the event network

### Identifying and sequencing the events

In order to code the events and their causal connections, theoretical/conceptual frames can be used, like the theoretical/conceptual table built from the set of eight elements proposed by Heise and Durig [21] - from now on referred to as “event frame” or “EF” (Table 1). Concerning other theoretical/conceptual frames used for the formal representation of events, EF has been considered a distinctively systematic semantics (c.f. [2]). These elements were identified from the work of Charles Fillmore on “linguistic cases” [11] as the set of basic meaning categories used by people to describe a social event in a whole way, whatever the language.

**Table 1:** Event Frame (EF).

Element	Definition
Agent	The instigator of a happening.
Action	The fusing of event-frame elements into a happening.
Object	The entity that is moved or changed, such that a repetition of the happening requires replacement. People can be objects.
Instrument	An entity that is used by the agent to causally advance the happening while not being significantly changed by the happening. People, social organizations, and verbalizations can be instruments.
Alignment	The specific place or time at which an instrument is applied to an object or in a setting.
Product	An entity that comes into existence as a result of a happening and that enables or disables subsequent happenings.
Affected^a^	The agent of an event that intentionally is enabled or disabled by the agent in the focal event.
Setting	A convergence of relatable agents, objects, and instruments within a space-time boundary.

a Term chosen to make clear that the “beneficiary” (original term in [21]) may be disabled by the agent of the focal event; that is, he/she might be, not a beneficiary, but a “victim” of the product of the action under analysis (c.f. [3]; Bergvall-Kåreborn, Mirijamdotter, & Basden, 2004). *Source:* Adapted from Heise and Durig [21].

### Linking events (inferring causal connections)

In order to infer causal connections between the events, the next step consists in the causal interpretation of the chronological sequence obtained. That is: for each pair of events, researchers must evaluate the possibility that the older event could be a cause of the more recent event. Based on this assessment, the existence or not of the corresponding causal connection is inferred (c.f. [26]).

The theoretical/conceptual frame suggested for coding causal connections between events relies on the notions of causal ``necessity'' and ``sufficiency'' (*e.g.*, [16,^31^,35]). Specifically, this study focused on necessary connections. After all, the inference of necessary causes has been considered by many the most feasible and desirable means of explanation in the social sciences [16]. Also, with rare exceptions, the inference of a connection as sufficient is risky, when dealing with historical processes - which means that, in general, this type of causality is reserved to the explanation of technical, and not social, processes [30].

In this context, “necessity” was associated with the counterfactual notion that a result would not have occurred if the cause were absent, although the presence of the cause does not guarantee the result. In set-theoretic terms, X is inferred as a necessary cause of Y if Y can be defensibly considered a subset of X (c.f. [31]) - that is, if, counterfactually, one could argue that there would not be any plausible historical situation in which an event of type Y (i.e. an event similar to the concrete outcome in analysis) would happen and an event of type X (i.e. an event similar to the potential cause in analysis) would not. To give a trivial but clear example, we may infer that sunlight is a necessary cause of rainbows, because - as far as we know (thus, an inference) - there is no plausible situation in which a rainbow could happen without sunlight.The inference of this type of causality, therefore, is not based on correlations, but on the so-called ``explicit'' or ``set-theoretic'' connections [34] – *i.e.,* connections that fit this implicative logic that can also be represented in set-theoretic terms.

This view of causation in terms of necessity and sufficiency has been considered more adequate to qualitative explanation (and to historical-comparative approaches, in particular), than the statistical outlook of ``cause as a leverage, on average, of the probability of a result” [28,31]. To infer the existence (or not) of this type of causality connecting one event to another in a particular case, two types of questions were used (Table 2).

**Table 2:** Type of question to be answered to infer if event X is a necessary cause of event Y in a case.

Type of causality	Implicative question	Counterfactual question
Necessary	Does the occurrence of Y imply the prior occurrence of an event similar^a^ to X?	Suppose an event similar to X did not occur. Can Y occur?
	*Answer corresponding to the inference of a causal connection*: Yes	*Answer corresponding to the inference of a causal connection*: No

a *I.e.* considered, in the culture of the natives, equivalent to the event under discussion - and may even be the event itself. *Source:* Adapted from Goertz & Starr [16] and Heise [25].

The implicative question requires the evaluation of the necessity of the occurrence of an event, given the occurrence of another. On the other hand, counterfactual questions demand the investigation of the implication of the hypothesis of non-occurrence of an event for the possibility of the occurrence of another. Both are logically equivalent, leading, in principle, to opposite answers - in terms of “yes” and “no” [25]. In order to infer that an event is necessary for another, in a particular case, the answer must be affirmative to the implicative question and negative to the counterfactual question (Table 2).

In order to consistently respond to these questionings, however, it is necessary to corroborate the position to be taken (*i.e.* the answer to the question) in specific and general aspects that apply to the connection being assessed. In other words, researchers must base their answers on specificities of the case and on evidence of comparable cases, relevant theories or other logical or common-sense generalizations [6,^7^,^12^,^14^,^18^,30]. This interaction between the particular and the general in the justification of causal interpretation is considered the essential component for the possibility of an effective historical explanation [18,^19^,30] and was, therefore, the focus of the authors in the attempt to respond to the implicative and counterfactual questions.

Supplementing these questions, the authors also recommend an adoption of the logic of process tracing tests (Collier, 2010, [9,^14^,^15^,30]) to analyze the hypothesis of the existence, in a particular case, of a causal connection between any two events. In the case of the logical test that uses a sufficient mechanism for the non-rejection of the hypothesis [31], it is established that the identification of a mechanism M, that is necessary for Y and requires X, is considered sufficient (but not necessary) to not reject the hypothesis that X is necessary for Y. Thus, all necessity connections in this paper were inferred based on this test. That is, for each assessed pair of events, the authors searched, through thought experiments, for an intermediary event (*i.e.* mechanism) that would plausibly be connected to both original events by necessity relationships. Of course, such a procedure could be deemed to incur into infinite regression, since these mechanism-related necessity connections themselves would also need to be tested. However, it is considered an acceptable methodological procedure to stop the recursion when the proposed mechanism relationships are intersubjectively obvious enough to be agreed upon as plausible, without further justification [30,31].

Gladly, though, not all pairs of sequential events have to be analyzed. Specifically, when the causal interpretation is carried out in terms of ``necessity'', certain causal connections can be logically deduced. After all, a necessary cause of a necessary cause of an event is a necessary cause of that event [23,31] – *i.e.* if A is a necessary cause of B and B, of C, then A is a necessary cause of C. Thus, these logical simplifications could be applied to some causal connections, making it unnecessary to evaluate them.

To support this process, the ESA software can be used [25] – *c.f.* (https://cs.uwaterloo.ca/~jhoey/research/ACTBackup/ESA/ESA.html). This program optimizes the sequencing of evaluations to be carried out by researchers, since it guides the process according to the chronology of events and to the possibility of logical simplification. It sequences the iterations taking into account the inferences made so far, in order to minimize the number of pairs of events to be assessed by researchers.

The ESA software also enables the recording, not only of the inferences made (*i.e.* if researchers supposed there was - or not - a causal connection), but also of the reasons on which these inferences were based. In this way, it is possible to recover the justifications for the causal structure obtained – which is an essential feature in order to submit the result to rational critiques [18,19]. Therefore, whenever possible, the mechanisms used to infer the necessary connection should also be recorded.

## Analyzing the network model

**Fig. 1:**
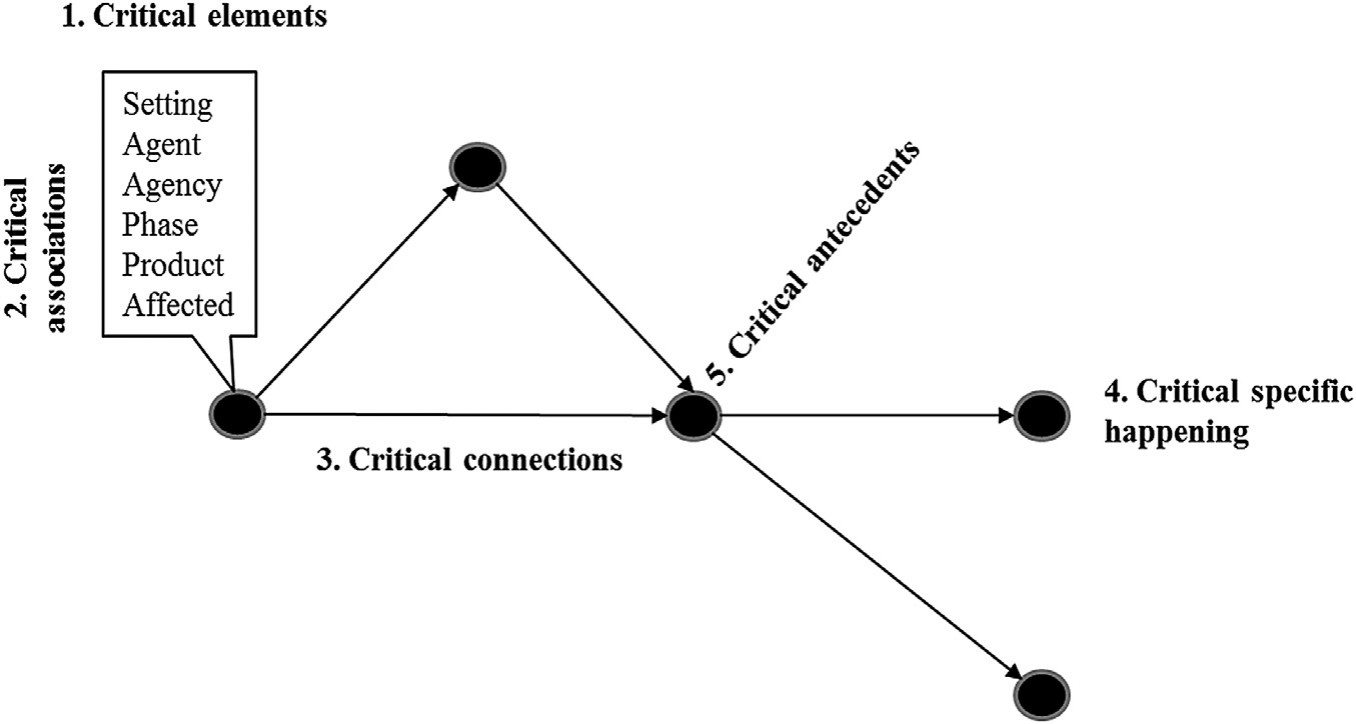
Main types of analysis for an event network.

*Source:* The authors.

Once this essential network model is built, it needs to be analyzed. Five main types of analysis can be carried out: identification of critical (i) elements; (ii) associations; (iii) connections; (iv) specific happenings; (v) and antecedents of these happenings. Fig. 1 visually summarizes these types of analysis.

In general, criticality can be initially assessed in terms of frequency of occurrence - *e.g.*, how many times a type of event (*i.e.* how many different concrete events that can be seen as instances of a same class of abstractly described event) happened. For example, each project milestone presentation to a top manager is a historically (*i.e.* “concretely”) unique event, but they are all occurrences of a same conceptual (*i.e.* “abstract”) event (*e.g.* “presenting results to top management”). However, in several analyses, besides the relative frequency of the event of interest (in relation to the total events), the following measures can be taken into account: the quantity of different components of the other element associated to the code under analysis (*e.g.* quantity of levels of analysis of “agent” associated to ``technological resource''). Thus, for instance, instances of three different analytical levels (e.g., individuals, groups, and organizations) may have been identified as agents who produced new technological resources as products of their actions in the historical process under analysis.

Also, four types of structural criticality are defined:

(i) Critical divergences - events whose outdegree (i.e. number of causal connections with subsequent events) is greater than a lower limit established from the outdegree distribution in the corresponding model. Thus, for example, one may consider an event that has an outdegree greater than the outdegrees of, say, 75% of the other events as a critical divergence.
(ii) Critical convergences - events whose indegree (i.e. number of causal connections with precedent events) is greater than a lower limit established from the indegree distribution in the corresponding model. Thus, similarly to the rationale used for inferring critical divergences, one may consider an event that has an indegree greater than the indegrees of 75% of the other events as a critical convergence.
(iii) Critical milestones - events defined both as a critical divergence and a critical convergence – that is, the degree (i.e. sum of indegree and outdegree) of which is greater than the lower limit established from the degree distribution (e.g. greater than, again, the 75-percentile) in the corresponding model; and
(iv) Critical intermediations - events whose betweenness - as defined by Wasserman and Faust [39] - is greater than the lower limit established from the betweenness distribution in the corresponding model. Betweenness is a network centrality measure that properly captures how central a network node (in our case, an event of the event structure) is in intermediating the flow (in our case, the “causal flow”) between all pairs of nodes in the network [39]. Thus, one may consider an event that has a centrality betweenness greater than the betweenness level of 75% of the other events as a critical intermediation. This last measure serves as an indicator of the cumulative (i.e. until the focal event) path dependence.

Based on these characterizations, inferences can be made about the types of events that can be more critical for the macro-outcome of the process (*i.e.* the phenomena of interest).

In all cases, from the evidence obtained, inferences may be proposed on ideal-typical behaviors expected to be observed in similar contexts. This form of “portability” of the results of a unique case is based on the analytical premise of ``thin rationality'' [4], according to which the social mechanisms found in a case can be carried over to other similar contexts, if conceived as ideal-typical expected patterns of action and interaction [4] – as in this research. Therefore, it is not assumed that the results are directly generalizable to another particular case, but to an imagined ``population'' of similar patterns in similar contexts [4].

### Example of application

We briefly present the basics of the application of Event Structure Analysis (ESA) in a causal case study about the process of capability construction for open innovation management in an Industrial Electronic Manufacturer (“IEM”). Melo et al. [32] present theoretical discussions about this case study. In order to identify and sequence the events, data was collected through participant observation for three years and refined by semi-structured cross-validating interviews with key stakeholders from IEM. The final event list is presented in Table 3.

**Table 3:** Event list.

#	Event (long name)	Event (encoded)	Agent	Agency
1	IEM associates to a French Engineering Company	EP1 ass GP1	Company	Associate
2	IEM changes its business model to provide turn-key solutions	EP1 cha bus mod	Company	Change business model
3	A Researcher (AGI-9) develops an integrated digital supervision, protection and control system (R&D-1)	AGI9 dev R&D1	Internal individual	Develop
4	IEM acquires a punching machine for the production process of electric panels	EP1 acq punc	Company	Acquire
5	IEM register the integrated digital supervision, protection and control system (R&D-1)’s brand in the National Institute of Industrial Property (INPI)	EP1 reg brand R&D1	Company	Register brand
6	Government sanctions an ``Innovation Law''	Gov sanc Inn Law	Government	Sanction
7	Top Management identifies funding opportunities for innovation	TM ide op fund	Top management	Identify opportunity
8	The Automation Department develops “Test Gigas Project”- a device for automatization of panel's final tests (R&D-3)	AD dev R&D3	Department/sector	Develop
9	Top management allocates an Innovation Manager (AGI-3) to lead the Innovation Center initiative (NGI)	TM alo AGI3 to (NGI)	Top management	Allocate
10	The Innovation Manager (AGI-3) present innovation projects to funding agencies	AGI3 pre proj fund	Internal individual	Present project
11	IEM implement ``ideation boxes''	EP1 imp idea box	Company	Implement
12	IEM approves the development of a high-performance microprocessor rectifier prototype (R&D-2) with a state-owned energy company	EP1 apr R&D2 PROG1 ENERG1	Company	Approve project
13	Top Management hires a new Innovation Manager (AGI-6) for innovation management (NGI)	TM acq AGI6 p NGI	Top management	Acquire
14	A Science and Technology Institute (ICT-2) makes partnership with IEM for the development of a software to increase the efficiency of hydroelectric generation (R&D-4) with a state-owned energy company	ICT2 mak par EP1 R&D4 PROG1 ENERG1	STI (Science and Technology Institute)	Make partnership
15	The Innovation Manager (AGI-6) perceives opportunity to frame “Test Gigas Project” (R&D-3) in government tax incentives program (PROG-5)	AGI6 ide op R&D3 PROG5	Internal individual	Identify opportunity
16	The Innovation Manager (AGI-6) makes a partnership with a Science and Technology Institute (ICT-3) for the development of a medium voltage panel (36kV) with reduced dimensions (R&D-5)	AGI6 mak par ICT3 des R&D5 PROG6	Internal individual	Make partnership
17	The Innovation Manager (AGI-6) approves new financial grants for the high performance microprocessor rectifier prototype (R&D-2), medium voltage panel (36kV) with reduced dimensions (R&D-5), and incremental improvements in columns of CCMs and panels of low voltage (R&D-10) projects	AGI6 apr FOM4 R&D2,5,10	Internal individual	Approve project
18	NGI approves financing for a platform of instrument transformers for high voltage (72.5 - 550kV) development (R&D-9)	NGI apr FOM1 R&D9	NGI	Approve project
19	NGI approves the development of a computational system for the management of medium and low voltage network assets (R&D-12) in partnership with a Science and Technology Institute (ICT-2)	NGI apr R&D12 ICT2 PROG7	NGI	Approve project
20	IEM presents ``solar photovoltaic energy generation'' project	EP1 pre proj solar	Company	Present project
21	Project team tests the medium voltage panel (36kV) prototypes abroad	TP test prot R&D5	Project team	Test prototype
22	Top management restructures IEM - creation of an innovation management department	TM ree EP1	Top management	Restructure
23	The Innovation Manager (AGI-6) leaves IEM	AGI6 leaves EP1	Internal individual	Leave company
24	NGI prepares a proposal for the development of a medium voltage panel composed of 2 (two) circuit breakers per column (R&D-45) with a Science and Technology Institute (ICT-3)	NGI pre proj R&D45 ICT3 PROG6	NGI	Present project
25	The Innovation Manager (AGI-19) leaves IEM	AGI19 leaves EP1	Internal individual	Leave company
26	IEM makes partnership with a government agency to develop new innovation management capabilities	EP1 mak par GOV4 PROG12	Company	Make partnership
27	"R&D Department" implements ``Visual management''	"R&D" imp Vis Mng	Department/sector	Implement
28	Top management allocates a new Innovation Manager (AGI-23) to lead ``R&D Department''	TM acq AGI23 R&D Dep	Top management	Acquire
29	Shareholders sell IEM to a French company	Shareholders sell EP1,4,5 GP7	Shareholders	Sell
30	"R&D Department" implements ``Supervision committee''	"R&D" imp Sup Comm	Department/sector	Implement
31	The Vice-President (AGI-1) leaves IEM	AGI1 leaves GP7	Internal individual	Leave company

For each event, some entities were coded. “AGI”, for instance, is an internal agent that was relevant to the outcome's historical background. The numbers after the codes (*e.g.* “AGI-1”, “AGI-2”) differ entities of the same category. Event #3 (“AGI9 dev R&D1”) represents the development of an integrated system for protection and control of power plants (encoded as “R&D-1”) led by an internal employee identified as “AGI-9”. Event descriptions have been shortened in number of characters (“Event - encoded” in Table 3) to serve as an input to the ETHNO Software (https://cs.uwaterloo.ca/~jhoey/research/ACTBackup/ESA/ESA.html). Table 3 also presents two elements per event, categorized using part of Heise and Durig [21]’s event frame (*i.e.* Agent and Agency).

The relationships (causal linkages between events) were inferred by using the questioning optimization algorithm of the ETHNO Software, choosing the counterfactual question for each pair of events prompted by the program (*i.e.* “Suppose that a similar event X doesn't occur. Can Y happen?”). In addition, causal mechanisms that justify the linkage between each pair of events were identified in a process tracing logic [30]. Exemplifying, the connection #25 (Table 4) shows the causal linkage between event #16 (AGI6 mak par ICT3 des R&D5 PROG6) and event #21 (TP test prot R&D5). Event #16 refers to a specific partnership carried out by an innovation manager (AGI6) with a Science and Technology Institute (ICT-3) for the development of a new electric panel (R&D-5) in the context of a national innovation program (PROG-6) which provided non-refundable financial resources for the winning projects. Event #21 refers to prototype tests performed by the R&D-5 project team to validate technical specifications of the new product. In sum, if the project was not initiated, prototype tests could not be performed. The mechanism “EP1 manufactures R&D5 prototypes” was created to reinforce this linkage (*i.e.* we assume it to be intersubjectively obvious that, if the project had not been initiated, prototypes could not have been manufactured – and if prototypes had not been manufactured, they could not have been tested).

**Table 4:** Mechanisms linking the most relevant events.

Connection ID	Events connected	Mechanism
1	1–2	IEM perceives opportunity to provide turn-key solutions
2	2–3	IEM hires a Researcher (AGI-9)
3	2–4	IEM increases solutions sales
4	3–5	IEM presents integrated digital supervision, protection and control system (R&D-1) for the National Institute of Industrial Property (INPI)
5	6–7	The Vice-President (AGI1) takes notice of the ``Innovation Law''
6	4–8	IEM increases electric panels production
7	7–9	Top Management realizes the need to allocate a specific employee for innovation management
8	9–10	The Innovation Manager (AGI-3) stimulates innovation idea generation in IEM
9	9–11	IEM realizes the need of a mechanism to collect ideas
10	7–12	Top Management encourages IEM's employees to submit internal projects to funding agencies
11	9–13	The Innovation Manager (AGI-3) leaves IEM
12	10–14	A Science and Technology Institute (ICT-2) approves the development of a software to increase the efficiency of hydroelectric generation (R&D-4) with a state-owned energy company
13	2–14	IEM provides solutions to ``Tres Marias'' power plant
14	13–15	The Innovation Manager (AGI-6) knows the ``Innovation Law'' - a government tax incentives program (PROG-5) - in a event
15	8–15	The Innovation Manager (AGI-6) studies ``Test Gigas'' (R&D-3) financial viability (after project closing)
16	13–16	The Innovation Manager (AGI-6) takes notice SENAI-SESI program (PROG6)
17	4–16	IEM increases electric panels production capacity
18	16–17	The Innovation Manager (AGI-6) presents microprocessor rectifier prototype (R&D-2), medium voltage panel (36kV) with reduced dimensions (R&D-5), and incremental improvements in columns of CCMs and panels of low voltage (R&D-10) projects to funding agencies
19	12–17	The Innovation Manager (AGI-6) presents microprocessor rectifier prototype (R&D-2), medium voltage panel (36kV) with reduced dimensions (R&D-5), and incremental improvements in columns of CCMs and panels of low voltage (R&D-10) projects to funding agencies
20	13–18	The Innovation Manager (AGI-6) realizes the opportunity to frame the platform of instrument transformers for high voltage (72.5 - 550kV) project (R&D-9) in a finance program
21	14–19	NGI invites Science and Technology Institute (ICT2) to participate in the development of a computational system for the management of medium and low voltage network assets (R&D-12) due the advances in the development of a software to increase the efficiency of hydroelectric generation (R&D-4)
22	13–19	The Innovation Manager (AGI-6) takes notice of a new program for innovation project financing (PROG7)
23	7–20	IEM identifies ``solar photovoltaic energy generation'' as a priority for the Brazilian government (PROG11)
24	2–20	IEM develops solutions for energy generation
25	16–21	IEM manufactures ``Panel 36kV'' (R&D-5) prototypes
26	16–22	Top Management recognizes ``Panel 36kV'' (R&D-5) as a case of success
27	15–22	Top Management recognizes benefits of the ``Innovation Law'' for the businesses
28	22–23	Top Management incorporates NGI as a unit of the IEM's Engineering Department
29	21–24	NGI finishes ``Panel 36kV'' (R&D-5) with success
30	23–25	A new Innovation Manager (AGI19) assumes NGI
31	22–26	The Innovation Manager (AGI19) realizes availability of internal structure to participate in an ``innovation management program'' (PROG12)
32	26–27	"R&D Department" knows ``Visual management'' from the ``innovation management program''
33	25–28	Top Management realizes the need to allocate a specific employee for innovation management
34	1–29	Shareholders create a bond with a French company
35	28–30	"R&D Department" knows ``Supervision Committee'' from the ``innovation management program''
36	26–30	"R&D Department" knows ``Supervision Committee'' from the ``innovation management program''
37	29–31	The Vice-President (AGI1) assumes an executive post in the French company

**Fig. 2:**
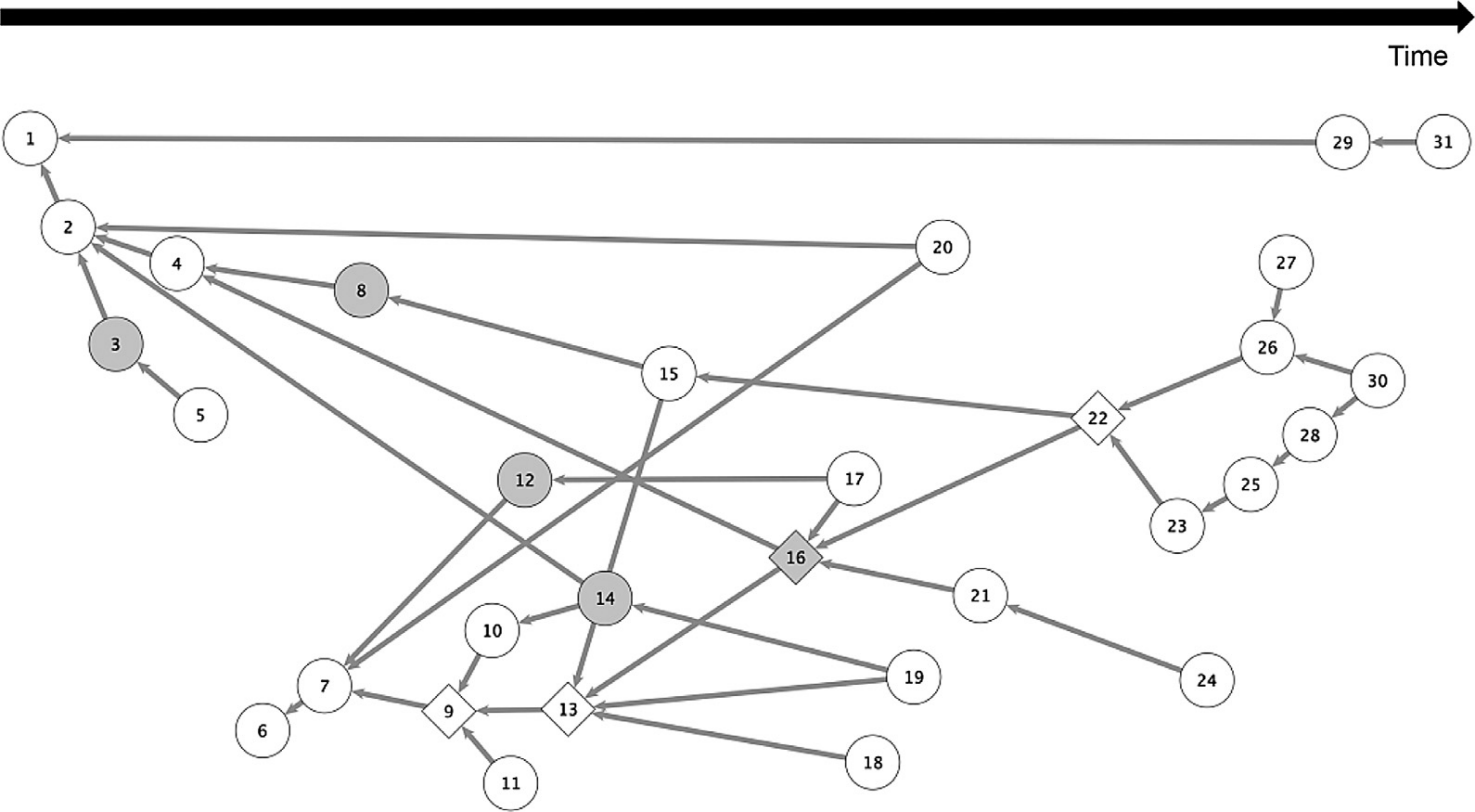
The causal event structure. *Notes:* (i) circles: typical events; (ii) diamonds: turning point events; (iii) grey circles/diamonds: events concerning main innovation projects; (iv) arrows: necessary causal connections between events, read as “the more recent event (i.e. in time) implies (i.e. logically/counterfactually) the older event”.

The causal event structure is presented in Fig. 2 – the 31 events considered the most important ones for the case are temporarily sequenced. This resulting network was modelled and analyzed using the VISONE Software (www.visone.info). Each event is represented through a circle with its respective codification. The arrows linking the circles are the causal connections between two distinct events.

Some visual effects in the network (*i.e.* diamonds, grey circles/diamonds) represent some results of the analyses that were carried out. Diamonds, for example, are events which were considered “critical specific happenings” for the story, meaning that if they were withdrawn from the network, the historical flux would have been interrupted. Gray symbols represent events in which the element “action” is the execution of an innovation project. This standardization was used in Melo et al*.* [32] to theoretically discuss the role of projects to build a new organizational capability. These insights were extracted from a “structural critically” analysis of the network, concerning the events (*e.g.* #9, #13, #16, #22) with the highest combination of the “degree” and “betweenness centrality” indices.

**Fig. 3:**
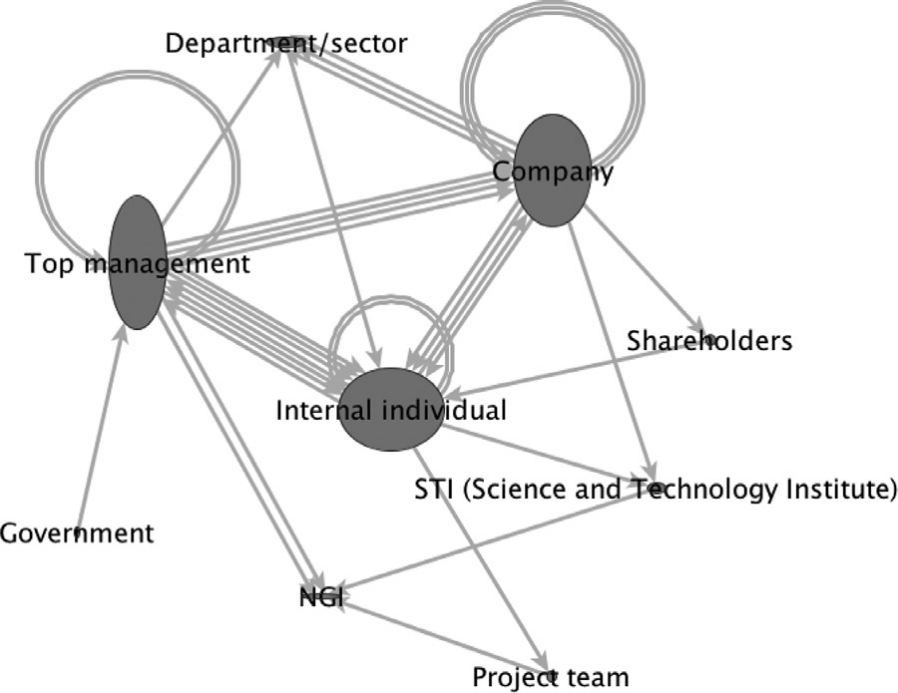
The most relevant agents in the story. *Notes:* (i) circle – type of agent; (ii) circle height – outdegree; (iii) circle width – indegree; (iv) arrows: an event instigated by the type of agent represented in the node at the tail of the arrow was inferred as causally necessary to an event instigated by the other type of agent represented in the head of the arrow.

Fig. 3 presents one illustrative example of “critical associations” representing a preliminary model of relationships between agents during the construction of an open innovation project management capability for the studied case. This model was constructed as follows. Firstly, we identified all the (abstractly defined) types of agents involved in the 31 events of our causal structure shown in Fig. 2. Each of the nine types of agents identified (Fig. 3) was, then, connected to another type of agent by an arrow if - and only if - there was, in our original causal structure, an event instigated by an instance of the first type of agent that was inferred as causally necessary to another event instigated by an instance of the second type of agent under consideration. If there were more than one pair of connected events instigated by the corresponding pair of types of agents, this number of original causal connections in Fig. 2 was represented by the number of arrows connecting the respective pair of agents in Fig. 3. Thus, for instance, as shown in Fig. 3, there was only one causal connection in our original event structure linking an event instigated by “top management” as a necessary cause of an event instigated by the “department/sector” type of agent. On the other hand, top manager(s) instigated four different events that were - each of them, individually - inferred as causally necessary to one of other four different events instigated by companies, respectively.

Therefore, in Fig. 3, the number of arrows between two circles represents the frequency with which the two corresponding nodes were connected as agents of two causally related events. Thus, it visually highlights the most and least frequent causal connections in the historical process in question. In this graphical representation, node width takes this information to represent the number of original events that led to the corresponding node, while node height represents the number of events that were caused by it. Hence, actions led by “Internal Individual” were more caused than causal while actions led by “Top Management” and “Company” were more causal than the opposite. Moreover, the relatively wide loop represented above the “Top Management”, “Company” and “Internal Individuals” nodes indicates that these agents frequently caused events initiated by other similar agents, pointing to some cumulative recursions in their interactions. It can also be noted that, at the core of this structure is the virtuous circle involving ``Top Management'', ``Internal Individual'' and ``Company'' - which can be considered the most influential actors in the story.

Analyses such as these may highlight some important processual patterns and exceptions that might not be noticed without such a systematic methodological procedure for modelling and analyzing the event structure. These, in turn, may, of course, help discussing theoretical propositions, their adherence or not to the case in question, and, specially, the possibilities of advancing previous knowledge on the basis of such a detailed micro-processual tracing of a macro-outcome of interest.

## Conclusions

Abstaining itself from discussing theoretical backgrounds or implications of its analyses, this paper presented a robust method to track the progression of a phenomenon over time in a truly processual approach. As such, it departed from the typical variance-based methodological paradigm, which, with rare exceptions (and at the expense of complicated adaptations), cannot adequately capture temporal flux - but, in general, limits itself to comparisons of static states over points in space or time. Taking temporality seriously, though, requires a shift from variables to events, and from abstract statistical regularities to case-based causal inferences of historical necessity.

However, this departure from conventional mainstream approaches does not degenerate into a purely narrative account, without any analytical potential. On the contrary, as this paper shows, rigorous inferences of dependences between events open up the opportunity to model a temporal sequence of events as a causal structure, which, in turn, may be submitted to various analyses in order to surface relevant abstractions from the causal flow. More specifically, through this inspection of the event network, a robust event coding scheme can be used to assess patterns and exceptions in terms of event elements, associations between these elements and connections between different events.

These historically grounded evidence may illuminate mechanisms intermediating event-related variables previously connected (in statistical terms) in the literature or, even, serve as a basis for new theoretical propositions of behavioral deployments over time that may be observed in similar contexts. Therefore, this innovative proposal on how to apply event structure analysis may contribute to supplement and enrich knowledge sharing practices in disciplines dealing with inherently processual phenomena. Specifically, for the engineering field, this adapted ESA method can support a wide range of organizational problems associated with complex engineering projects which may involve long causality chains within a project and/or high level of path-dependence among projects. The example we discuss in this paper is focused on project/organization levels of analysis, but there is enormous potential for future studies in engineering or other tech-intensive settings where one could apply the method to get insights at other levels of analysis.

## Declaration of Competing Interest

The authors declare that they have no known competing financial interests or personal relationships that could have appeared to influence the work reported in this paper.

## References

[1] Abbott A. (1995). Sequence analysis: new methods for old ideas. Annu. Rev. Sociol..

[2] Abell P. (2004). Narrative explanation: an alternative to variable-centered explanation?. Annu. Rev. Sociol..

[3] Basden A., Wood-Harper A.T. (2006). A philosophical discussion of the root definition in soft systems thinking: an enrichment of CATWOE. Syst. Res. Behav. Sci..

[4] Bengtsson B., Hertting N. (2013). Generalization by mechanism: thin rationality and ideal-type analysis in case study research. Philos. Soc. Sci..

[5] Bennett A., Checkel J.T. (2012). Process Tracing in the Social Sciences: From Metaphor to Analytic Tool.

[6] Bennett A. (2006). Stirring the frequentist pot with a dash Bayes. Polit. Anal..

[7] Bennett A., Box-Steffensmeier J., Brady H.E., Collier D. (2008). Process tracing: a Bayesian perspective. The Oxford Handbook of Political Methodology.

[8] Blatter J., Haverland M. (2012). Designing Case Studies: Explanatory Approaches in Small-N Research.

[9] Collier D. (2011). Understanding process tracing. PS: Polit. Sci. Polit..

[10] Corsaro W., Heise D. (1990). Event structure models from ethnographic data. Sociol. Methodol..

[11] Dirven R., Radden G. (1987). Fillmore's Case Grammar: A Reader.

[12] Freitas J.S., Gonçalves C.A., Cheng L.C., Muniz R.M. (2013). Structuration aspects in academic spin-off emergence: a roadmap-based analysis. Technol. Forecast. Soc. Change.

[13] Geertz C. (1973). The Interpretation of Cultures.

[14] George A.L., Bennett A. (2005). Case Studies and Theory Development in the Social Sciences.

[15] George A.L., McKeown T.J. (1985). Case studies and theories of organizational decision making.

[16] Goertz G, Starr H., Goertz G., Starr H. (2003). Introduction: necessary condition logics, research design, and theory. Necessary Conditions: Theory, Methodology, and Applications.

[17] Griffin L. (1992). Temporality, events, and explanation in historical sociology: an introduction. Sociol. Res. Methods.

[18] Griffin L. (1993). Narrative, event-structure analysis, and causal interpretation in historical sociology. Am. J. Sociol..

[19] Griffin L.J., Korstad R.R. (1998). Historical inference and event-structure analysis. Int. Rev. Soc. Hist..

[20] Griffin L.J., Ragin C.C. (1994). Some observations on formal methods of qualitative analysis. Sociol. Methods Res..

[21] Heise D.R., Durig A. (1997). A frame for organizational actions and macroactions. J. Math. Sociol..

[22] Heise D.R., Lewis E. (1988). Program Ethno.

[23] Heise D.R. (1989). Modeling event structures. J. Math. Sociol..

[24] Heise D.R., Fielding Nigel, Lee Raymond (1991). Event structure analysis: a qualitative model of quantitative research. Using Computers in Qualitative Research.

[25] Heise D.R. (2012). *Program ESA: Manual*.

[26] Hodgkinson G.P., Maule A.J., Bown N.J. (2004). Causal cognitive mapping in the organizational strategy field: a comparison of alternative elicitation procedures. Org. Res. Methods.

[27] Kittel B., Kuehn D. (2012). Eur. Polit. Sci..

[28] Mahoney J., Goertz G. (2006). A tale of two cultures: contrasting quantitative and qualitative research. Polit. Anal..

[29] Mahoney J. (2000). Strategies of causal inference in small-N analysis. Sociol. Methods Res..

[30] Mahoney J. (2012). The logic of process tracing tests in the social sciences. Sociol. Methods Res..

[31] Mahoney J., Kimball E., Koivu K. (2009). The logic of historical explanation in the social sciences. Comp. Polit. Stud..

[32] Melo J.C.F.de, Salerno M.S., Freitas J.S., Bagno R.B., Brasil V.C (2020). From open innovation projects to open innovation project management capabilities: a process-based approach. Int. J. Project Manag..

[33] Mohr L.B. (1982). Explaining Organizational Behavior.

[34] Ragin C.C., Rihoux B. (2004). Qualitative comparative analysis (QCA): state of the art and prospects. Qual. Methods. Newsletter Am. Polit. Sci. Assoc. Org. Sect. Qual. Methods.

[35] Ragin C.C. (2000). Fuzzy-Set Social Science.

[36] Stevenson W.B., Greenberg D. (1998). The formal analysis of narratives of organizational change. J. Manag..

[37] Stevenson W.B., Greenberg D. (2000). Agency and social networks: strategies of action in a social structure of position, opposition, and opportunity. Admin. Sci. Q..

[38] Valorinta M., Schildt H., Lamberg J. (2011). Path dependence of power relations, path-breaking change and technological adaptation. Ind. Innovat..

[39] Wasserman S., Faust K. (1994). Social Network Analysis: Methods and Applications.

